# Preliminary Findings from a Mindfulness-Based Intervention in Patients with Psychogenic Non-Epileptic Seizures

**DOI:** 10.3390/neurolint17100171

**Published:** 2025-10-14

**Authors:** Rebecca Ciacchini, Ciro Conversano, Graziella Orrù, Chiara Pizzanelli, Claudia Scarpitta, Francesco Turco, Enrica Bonanni, Annachiara Bressan, Thomas Reali, Angelo Gemignani

**Affiliations:** 1 Department of Surgical, Medical and Molecular Pathology, Critical and Care Medicine, University of Pisa, 56010 Pisa, Italy; 2 International School of Advanced Studies, University of Camerino, 62032 Camerino, Italy; 3 Neurology Unit, Department od Clinical and Experimental Medicine, University of Pisa, 56010 Pisa, Italy

**Keywords:** psychogenic non-epileptic seizures, mindfulness, mindfulness-based intervention, MBI, emotional regulation, interoception, dissociation, sleep quality

## Abstract

**Background/Objectives**: Psychogenic non-epileptic seizures (PNES) are seizure-like episodes not caused by abnormal brain activity, often linked to emotional dysregulation, dissociation, and altered interoceptive awareness. Standardized treatments are limited. This study aimed to explore the feasibility and preliminary psychological effects of a group-based mindfulness-based intervention (MBI) in individuals with PNES. **Methods:** This single-arm, pre–post pilot study (no control group) enrolled fifteen participants in two cycles of an 8-week MBI delivered either in-person or online. Twelve participants completed pre/post self-report assessments of depression (BDI-II), anxiety (STAI-Y1), perceived stress (PSS-10), sleep quality (PSQI), dissociation (DES-II), meteoropathy (METEO-Q), mindfulness (FFMQ), and interoceptive awareness (MAIA). **Results:** The intervention was well tolerated (dropout rate: 20%). Trend-level, non-significant improvements emerged for depressive symptoms (*p* = 0.092, r = 0.564) and sleep quality (*p* = 0.078, r = 0.591). A significant reduction was observed in the FFMQ Describing subscale (*p* = 0.045, r = 0.697). No significant changes were found in anxiety, perceived stress, or interoceptive awareness, although certain MAIA subscales indicated small, non-significant increases. **Conclusions:** Despite the limited sample size and absence of a control group, these preliminary findings support the feasibility and acceptability of MBIs for PNES, warranting further controlled investigations.

## 1. Introduction

Functional seizures, also known as psychogenic non-epileptic seizures (PNES), are defined as paroxysmal events characterized by motor, sensory, autonomic, and/or cognitive alterations that mimic epileptic seizures but occur without concomitant epileptiform activity [1,2]. Despite growing attention in recent years, PNES remain among the most underdiagnosed and clinically misunderstood conditions within the spectrum of functional neurological disorders. This condition is associated with significant levels of personal distress, frequent medical consultations, and considerable social and occupational impairment [3]. In many cases, patients are unable to drive and refrain from going out alone, not only due to fear of physical harm during an episode, but also because of the fear of others’ judgement [3]. Despite the considerable burden on daily functioning and quality of life, standardized diagnostic and therapeutic approaches are still lacking; these shortcomings frequently result in delayed or missed diagnoses and fragmented care pathways, which in turn can negatively affect long-term outcomes [4].

Epidemiological data suggest a prevalence of 2 to 33 cases per 100,000 individuals, with PNES accounting for approximately 20–30% of patients referred to epilepsy centers [5]. The condition appears to be more common among women and is frequently associated with psychiatric comorbidities such as post-traumatic stress disorder (PTSD), anxiety, depression, and a history of childhood trauma [6]. Neurobiological and neuroimaging studies have identified functional alterations in brain regions involved in emotion regulation, interoception, and motor control [7,8]. In addition, individuals with PNES often show dissociative tendencies, alexithymia (i.e., difficulties recognizing and verbalizing emotions), emotional avoidance, and impaired sense of agency, suggesting a multifactorial and heterogeneous pathophysiology [9,10,11,12].

Several theoretical models have been proposed to explain PNES, including trauma-related dissociation frameworks, the Integrative Cognitive Model (ICM), and neurobiological perspectives emphasizing emotion dysregulation and altered sense of agency [3,12,13]. These models highlight the interplay of predisposing, precipitating, and perpetuating factors, with particular focus on dissociative mechanisms, difficulties in emotion regulation, and deficits in the integration of cognitive and affective processes. According to the ICM and the emotional–cognitive vulnerability hypothesis, PNES may arise as maladaptive learned responses to stress, shaped by prior trauma, affect suppression, and impaired executive control [3]. Neuroimaging findings further support these conceptualizations by demonstrating altered connectivity in circuits involved in emotion processing, sensorimotor integration, and attentional control [7,8].

In the effort to identify effective treatments for PNES, multi-component interventions combining psychoeducation, cognitive-behavioral strategies, and coping skills development have shown promising results in reducing seizure frequency, although broader psychosocial outcomes remain underexplored [14]. Among emerging non-pharmacological approaches, mindfulness-based interventions (MBIs) have gained attention for their capacity to target core mechanisms of the disorder. Mindfulness practice, commonly described as the intentional and non-judgmental awareness of present-moment experiences, has demonstrated beneficial effects on emotion regulation, attentional control, and stress-related physiological reactivity [15,16,17]. MBIs are structured programs that integrate formal meditation practices (e.g., body scan, sitting meditation, mindful movement) with psychoeducation and group discussion, with Mindfulness-Based Stress Reduction (MBSR) [18,19] being the best-established model, being an 8-week structured program that combines individual daily practice with weekly group sessions. Evidence from clinical research indicates that MBIs can reduce anxiety, depression, and dissociation, while enhancing stress resilience and interoceptive processing—dimensions frequently impaired in PNES [20,21,22]. At the neurophysiological level, mindfulness meditation has been shown to modulate intracranial EEG activity across epileptic and non-epileptic brain regions, suggesting a potential impact on dysregulated circuits implicated in PNES pathophysiology [23]. More broadly, mindfulness practice has been found to influence key psychological and neurobiological processes, including improved emotion regulation, reduced rumination, heightened interoceptive awareness, and decreased experiential avoidance [24,25].

MBIs may represent a particularly suitable therapeutic approach, as they directly target the aforementioned transdiagnostic vulnerabilities by fostering present-moment awareness, cultivating non-judgmental acceptance, and strengthening emotional regulation [see Table 1].

## 2. Materials and Methods

### 2.1. Aims and Context

The present pilot study aims to provide preliminary evidence on the effects of an MBI program in individuals diagnosed with PNES. Considering the scarce and still developing body of research on mindfulness-based interventions for patients with PNES—most of which relies on individualized treatment formats, such as those proposed by Baslet and colleagues—we chose to adopt the group-based approach [26].

As highlighted in the previous cited studies, patients with PNES are often referred from one specialist to another—neurologists, psychiatrists, psychologists—without a coordinated treatment plan or effective communication among providers. This fragmentation of care reflects a broader issue observed in other chronic conditions, such as chronic pain, in which patients are repeatedly cycled through services without integrated support [10]. In this context, the present study was initiated following a request from the Neurology Unit of the Pisa University Hospital (AOUP), which identified the need for psychological interventions targeting stress reduction and emotional regulation in PNES patients. These individuals often fall through the cracks of the healthcare system, where diagnosis alone is not sufficient to ensure appropriate therapeutic support.

Although the intervention was developed and implemented in a university setting rather than as part of routine hospital care, it represents a first step toward the establishment of a structured and replicable treatment model. As the aim of the present study, we evaluated the impact of mindfulness training on psychological dimensions including depressive symptoms, anxiety, sleep quality, dissociation, perceived stress, and interoceptive awareness. Moreover, in light of the multifactorial etiology of PNES and the theoretical alignment between its core features and mindfulness-based mechanisms, we hypothesized that participation in the MBI would be associated with improvements in emotional well-being and stress-related outcomes.

### 2.2. Study Design, Recruitment and Procedures

This is a feasibility study following a pre–post design without a control group. In the initial part of the study, participants were recruited at the Neurology Unit (UOC Neurology, Department of Neurosciences) of the University Hospital of Pisa (AOUP), within the framework of a University of Pisa research protocol (University of Pisa bioethics committee, resolution no. 54/2024). The diagnostic pathway included clinical interviews, neurological examinations, and at least one video-electroencephalographic (video-EEG) monitoring session.

Recruitment occurred during the final diagnostic consultation, in which the diagnosis of psychogenic non-epileptic seizures (PNES) was communicated by the attending neurologists and a psychologist. When appropriate, patients were invited to attend a separate psychological consultation. During this meeting, an unstructured clinical interview was conducted to assess eligibility. If considered suitable, patients were invited to participate in the MBI. Participation was entirely voluntary, and written informed consent was obtained prior to enrollment. The intervention recruitment was delivered in a university research setting and was not part of routine clinical care.

Inclusion criteria included: (1) age ≥ 18 years; (2) confirmed diagnosis of PNES based on video-EEG or multidisciplinary consensus; (3) adequate cognitive and linguistic abilities to engage in the intervention and complete questionnaires, assessed through the unstructured clinical interview and (4) sufficient proficiency in the Italian language. Exclusion criteria were: (1) presence of severe psychiatric conditions not adequately stabilized by pharmacological treatment—such as active psychosis or acute affective symptoms—assessed through clinical interview and (2) any condition involving significant psychological distress, severe social withdrawal, or behavioral patterns incompatible with group-based participation.

Selected participants were invited to attend an initial meeting with the mindfulness instructor, scheduled within two months of the diagnosis. They completed a battery of self-report questionnaires, which are explained in detail in the measures section, at two time points: between the orientation session (T0) and the start of the intervention, and again within one week after the final session (T1). Completion time for the full assessment was approximately 30 min.

Psychological assessments were administered online using secure digital platforms (Microsoft Forms) [27]. Participants received clear instructions on how to complete the questionnaires independently. In addition to self-report instruments, a routine clinical interview was conducted by experienced clinicians to collect information on PNES history, psychiatric and medical comorbidities, and ongoing treatments (See Appendix A). This was used to complement the clinical characterization of the sample. Seizure frequency was assessed by the neurologists through patient self-report, with participants asked to indicate the number of PNES episodes experienced during the previous month. Clinical data was also obtained from patient records with their consent. General technical and procedural support was available via email or telephone, and assistance was also offered in person when needed. The orientation session included a brief psychoeducational overview of mindfulness and stress. Confidentiality was ensured, and no financial compensation was provided.

### 2.3. Intervention

The intervention was primarily based on Jon Kabat-Zinn’s standard Mindfulness-Based Stress Reduction (MBSR) protocol [19], but it was adapted to meet the clinical and logistical needs of patients with PNES. To reflect these modifications, we refer to it as a Mindfulness-Based Intervention (MBI). Given participants’ reduced attentional capacity, each session was limited to a maximum of 120 min, and mindfulness exercises were initially kept very brief (starting at 3 min), with a gradual increase in duration over time. This flexible approach aimed to enhance adherence and engagement, while maintaining fidelity to the core principles of mindfulness practice. No yoga sessions were included, as these were considered logistically impractical. The full-day silent retreat was not administered.

The intervention was delivered in two separate cycles during 2024, each consisting of eight weekly group sessions with at least six participants enrolled at the start (first group: 9 participants; second group: 6. The online format (second group) was introduced in response to participants’ requests, as some reported mobility limitations or difficulties attending in-person sessions, highlighting the flexibility and accessibility of the protocol. Sessions were conducted by a licensed psychologist and psychotherapist with certified training in mindfulness (Author RC). Each session included core MBI components such as formal mindfulness practices (e.g., body scan, sitting meditation, mindful movement), experiential exercises, inquiry-based dialogue, and thematic discussions related to stress reactivity, emotional regulation, and mindful coping. Participants were encouraged to engage in daily home practice, progressively increasing from 15 to 45 min per day, using audio recordings and written materials provided by the instructor. Group discussions emphasized personal experience and reflection, while respecting individual privacy and group safety. Participants were encouraged to maintain any ongoing treatments as usual. Attendance at a minimum of six sessions was required to be included in the final analysis.

### 2.4. Measures

Participants completed a battery of self-report questionnaires at baseline and post-intervention to assess key psychological domains relevant to PNES, in the context of the MBI:

- Five Facet Mindfulness Questionnaire (FFMQ) [28]. FFMQ is a 39-item scale measuring five facets of mindfulness: observing, describing, acting with awareness, nonjudging, and nonreactivity. The Italian version of the FFMQ has demonstrated satisfactory psychometric properties in both clinical and non-clinical populations. In particular, the validation study confirmed the original five-factor structure through confirmatory factor analysis and reported good internal consistency (Cronbach’s α ranging from 0.72 to 0.86 across subscales) [29].
- Perceived Stress Scale (PSS-10) [30]. The PSS-10 is a widely used 10-item self-report questionnaire designed to measure the degree to which individuals perceive their lives as unpredictable, uncontrollable, and overloaded. The Italian version (IPSS-10) supported a two-factor structure—Perceived Helplessness and Perceived Self-Efficacy—and demonstrated good internal consistency (Cronbach’s α = 0.87). Convergent validity was confirmed through significant correlations with anxiety and depression measures, supporting the scale’s suitability for use in both research and clinical settings [31].
- Beck Depression Inventory-II (BDI-II) [32,33]. The BDI-II is a 21-item self-report inventory developed to assess the presence and severity of depressive symptoms over the past two weeks, in accordance with DSM-IV criteria. The Italian adaptation confirmed the robust psychometric properties of the instrument in Italian samples; high internal consistency was reported (Cronbach’s α = 0.80–0.91) across both clinical and non-clinical populations, as well as good convergent validity with other measures of depression and anxiety. The questionnaire demonstrated satisfactory discriminant validity, effectively distinguishing between clinical and non-clinical groups [33].
- State-Trait Anxiety Inventory—State version (STAI-Y) [34]. The STAI-Y is a 40-item self-report questionnaire designed to assess the intensity of anxiety experienced in the present moment and in general. The Italian version has shown good psychometric properties [35]. The STAI-Y1 was used for this study (state anxiety).
- Pittsburgh Sleep Quality Index (PSQI) [36]. The PSQI is a widely used 19-item self-report questionnaire designed to assess subjective sleep quality and disturbances over a one-month interval. It has a global score derived from seven component scores: subjective sleep quality, sleep latency, sleep duration, habitual sleep efficiency, sleep disturbances, use of sleep medication, and daytime dysfunction. The Italian validation confirmed the original structure and psychometric robustness of the scale, reporting good internal consistency (Cronbach’s α = 0.83). Notably, the study supported the established cut-off score of 5 to distinguish good sleepers from poor sleepers [37].
- Dissociative Experiences Scale—II (DES-II) [38]. The DES-II is a 28-item self-report instrument to assess the frequency of dissociative phenomena in everyday life, including amnesia, depersonalization, derealization, absorption, and identity confusion. Though not diagnostic, the scale serves as a screening tool for clinically relevant dissociative symptoms and is commonly used to identify individuals who may require further evaluation (e.g., via the SCID-D). The Italian version has been used in both clinical and non-clinical populations, particularly in studies involving trauma, functional neurological disorders, and personality pathology, confirming the scale’s sensitivity and clinical relevance [39].
- Multidimensional Assessment of Interoceptive Awareness (MAIA) [40]. The MAIA is a 32-item self-report instrument designed to assess interoceptive awareness across eight distinct dimensions: Noticing, Not-Distracting, Not-Worrying, Attention Regulation, Emotional Awareness, Self-Regulation, Body Listening, and Trusting. The MAIA was specifically developed for use in mind–body practices and has been widely applied in mindfulness-based research. The Italian version of the MAIA has shown good psychometric properties [41].
- Meteoropathy Questionnaire (METEO-Q) [42] was administered at baseline to assess meteorosensitivity and meteoropathy. It is a validated self-report tool composed of 11 core items (5 quantitative, 6 qualitative) and a checklist of 21 symptoms rated on a 0–4 Likert scale. The validation study confirmed satisfactory psychometric characteristics and provided percentile-based cut-offs distinguishing different levels of meteorosensitivity and meteoropathy [42].

### 2.5. Statistical Analysis

Due to the small sample size and the non-normal distribution of variables (as confirmed by the Shapiro–Wilk test), non-parametric statistical methods were employed. Specifically, the Wilcoxon signed-rank test for paired samples was used to assess pre–post differences in psychological and sleep-related outcomes. Descriptive statistics (mean, median, standard deviation, minimum, and maximum) were calculated for each variable at baseline and post-intervention. The threshold for statistical significance was set at *p* < 0.05. Results with *p* values between 0.05 and 0.10 were considered indicative of a statistical trend. Results should be interpreted as preliminary and hypothesis-generating. All analyses were performed using JASP software, version 0.19.3. [43].

## 3. Results

### 3.1. Sample Characteristics

**Figure 1:**
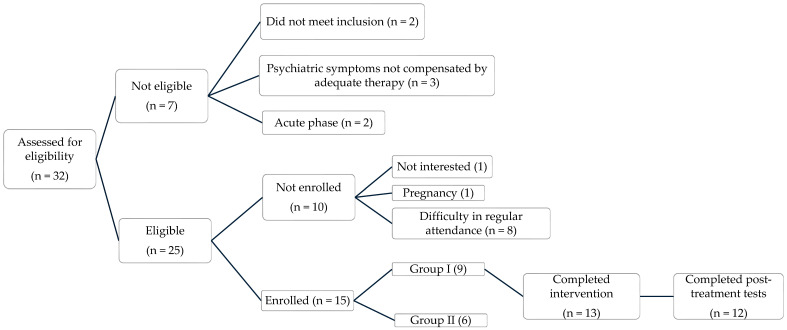
Flowchart of patient recruitment, eligibility, enrollment, and study completion.

A total of 32 individuals diagnosed with psychogenic non-epileptic seizures (PNES) were initially screened by neurologists and psychologists. Of these, 15 were enrolled in the intervention: the first cycle involved 9 participants scheduled to attend the in-person MBI program, while the second cycle included 6 participants assigned to the fully online format. Twelve participants (8 from the in-person group and 4 from the online group) completed both the intervention and the full set of assessments, resulting in a dropout rate of 20% (n = 3). No major feasibility differences were observed between delivery formats: eight of nine participants (89%) in the in-person group and four of six participants (67%) in the online group completed the program and post-assessment. Figure 1 displays the flowchart of patient recruitment, eligibility, enrollment, and study completion. 

Clinical information was obtained from the 15 patients’ medical records. The mean age of PNES onset was 39.8 years, with diagnosis occurring on average at 49.1 years. A documented history of epilepsy was present in 3 participants, while 11 reported none. Most participants presented psychiatric comorbidities, primarily anxiety (6) and depressive disorders (2) and some reported somatic conditions such as migraine (n = 8), obstructive sleep apnea (n = 2), or hypertension (n = 3). At study entry, some participants were receiving psychotropic or antiepileptic medications (9). Socio-demographic characteristics of the twelve patients who completed the intervention are presented in Table 2.

### 3.2. Baseline Clinical Characteristics

Baseline data were summarized through both descriptive statistics (means, standard deviations, and ranges) and clinical classifications derived from validated cut-off scores of the Italian versions of the questionnaires. As measured at baseline, participants exhibited clinically relevant levels of psychological distress when considering multiple domains commonly associated with PNES. Sleep quality was noticeably impaired in the majority of the sample: in fact, 9 participants scored above the clinical cut-off of 5 on the Pittsburgh Sleep Quality Index (PSQI), while only 1 scored below, and 2 were at the threshold. The meteoropathy questionnaire (METEO-Q), administered only at baseline, indicated high meteorosensitivity and meteoropathy levels in most participants, with 8 scoring in the high-risk range and 3 in the pathological range; only one individual fell within the intermediate range. In our sample, baseline scores on the dissociation levels (DES-II) were elevated in one participant, while others remained below the clinical threshold. Interoceptive awareness (MAIA) showed variability across its eight subscales; overall, participants tended to report lower scores in domains such as “Not-Distracting” and “Trusting”. Scores on the depression scale (BDI-II) showed 5 participants with minimal depressive symptoms, 4 reporting mild depression, and 3 with a score in the moderate range. Similarly, state anxiety levels (STAI-Y1) were elevated, though this measure does not have an established clinical cut-off. Perceived stress (PSS-10) resulted moderate in most participants (n = 8), while 2 individuals scored in the high range and 2 in the low range. Finally, baseline mindfulness scores on the Five Facet Mindfulness Questionnaire (FFMQ) were generally low, particularly in the “Acting with Awareness” and “Nonjudging” subscales, consistent with previously reported profiles in PNES patients.

### 3.3. Pre–Post Comparisons

Following the eight-week MBI, participants exhibited variable patterns of change across psychological domains [Table 3].

**Figure 2:**
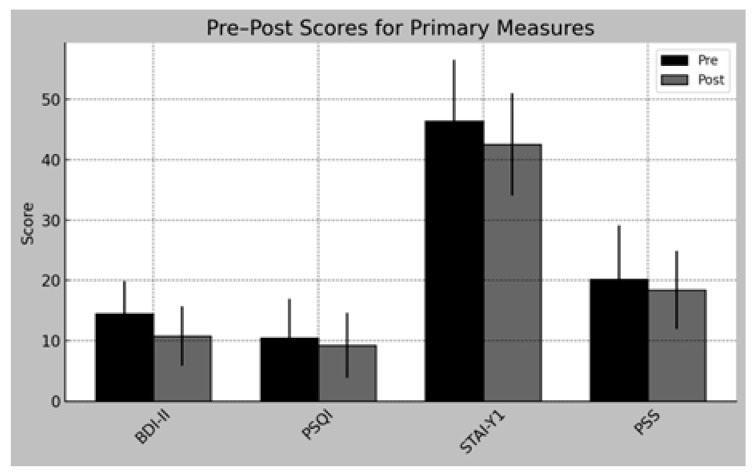
Pre–post intervention scores for primary psychological measures (BDI-II, PSQI, STAI-Y1, and PSS-10). Bars represent means with standard deviations. None of the outcomes reached statistical significance (*p* < 0.05); preliminary, non-significant trends were observed for depressive symptoms (BDI-II) and sleep quality (PSQI).

Although most outcomes did not reach statistical significance at the conventional threshold of *p* < 0.05, some variables showed non-significant trend-level improvements (*p* = 0.05–0.10), supported by moderate to large effect sizes. The “Dir” column indicates whether post-test values increased (↑) or decreased (↓) compared to pre-test values. (Figure 2).

Although most outcomes did not reach statistical significance at the conventional threshold of *p* < 0.05, some variables showed trend-level improvements (BDI-II, PSQI; *p* = 0.05–0.10), supported by moderate effect sizes. A significant reduction was observed in the FFMQ Describing subscale (*p* = 0.045), while the FFMQ total score and other mindfulness facets did not reach significance, despite some showing moderate effect sizes (e.g., Acceptance). Similarly, no significant changes emerged for state anxiety, perceived stress, or interoceptive awareness, though certain MAIA subscales (e.g., Self-Regulation, Trusting) indicated moderate, non-significant effects. Detailed statistics are reported in Table 3.

### 3.4. Change in Clinical Classifications

In addition to continuous score analyses, we explored clinically meaningful changes based on standard cut-offs for selected psychological measures. For the PSQI, the number of participants classified as “good sleepers” increased from 3 (25%) to 4 (33%) after the intervention, with a slight reduction in the “poor sleep” category. Depressive symptom severity, based on BDI-II categories, showed a shift from mild and moderate to minimal depression: 5 participants (42%) were in the “minimal” range at baseline, compared to 8 (67%) after the intervention. Finally, PSS-10 scores reflected a reduction in high stress cases (from 2 [17%] to 0), with most participants remaining in the moderate range across both time points.

### 3.5. Seizure Frequency (Exploratory Analysis)

**Figure 3:**
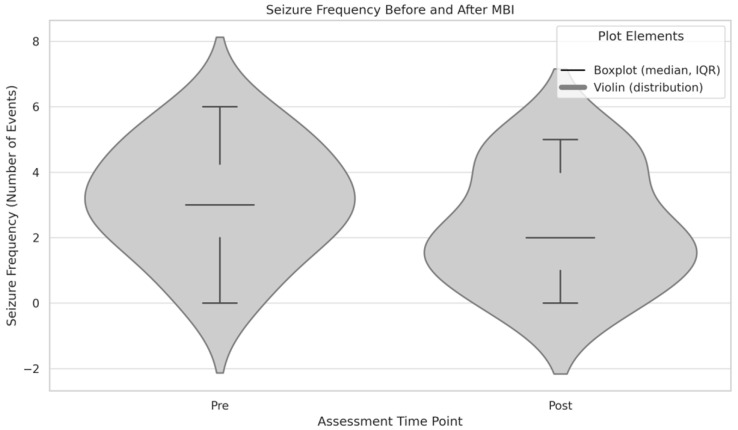
Distribution of seizure frequency before and after the mindfulness-based intervention (MBI). Violin plots display individual values, median, and interquartile range (IQR). The reduction in seizure frequency did not reach statistical significance (*p* = 0.169).

Although seizure frequency was not included among the primary outcome measures, exploratory analysis was conducted based on self-reported data. Given the small sample size and non-normal distribution of seizure frequency values (Shapiro–Wilk test: W = 0.805, *p* = 0.011), a Wilcoxon signed-rank test was performed to assess pre–post differences. The analysis revealed a non-significant reduction in seizure frequency following the intervention (Z = 1.468, *p* = 0.169), falling below both the conventional and trend-level thresholds for statistical significance [Figure 3]. Nonetheless, descriptive statistics indicated a decrease in the mean number of seizures from 3.00 (SD = 1.65) at baseline to 2.00 (SD = 1.71) post-intervention, suggesting a potential clinical relevance to be explored in future studies.

Lastly, qualitative feedback collected during debriefing indicated that while most participants engaged well with the program, some reported difficulties with the technical language of the meditations and with sustaining regular home practice. Others emphasized the supportive value of group sharing, though a few noted that the hospital setting felt impersonal.

## 4. Discussion

First, some methodological considerations should be addressed before discussing the findings. This study was a single-arm, pre–post feasibility pilot with a small clinical sample, and the findings are preliminary and hypothesis-generating. They must be interpreted with caution, as causal inferences about intervention effects are not possible without a control group. Observed improvements may reflect non-specific factors, and adequately powered randomized controlled trials—ideally multicenter—are needed to establish efficacy.

The two aims of the study were (1) to explore the feasibility and (2) preliminary psychological effects of an MBI in individuals diagnosed with psychogenic non-epileptic seizures (PNES). Despite growing interest in non-pharmacological approaches to PNES, empirical evidence remains limited, and mindfulness-based protocols have only recently been applied to this patients. In this study, an adapted program was administered to a small sample of PNES patients, with the goal of targeting common transdiagnostic vulnerabilities such as affective dysregulation, experiential avoidance, impaired interoception, and low mindfulness-related skills. While the small sample size limited the statistical power of our analyses, trends toward improvement in several psychological domains emerged, supporting the potential clinical relevance of mindfulness training for patients with PNES.

Even though no statistically significant results were found at the conventional threshold of *p* < 0.05, trends emerged for depressive symptoms and sleep quality, consistent with previous findings on mindfulness-based approaches in patients with PNES [22]. Most participants reported reductions in BDI-II scores, while PSQI scores indicated modest improvements, particularly in components related to sleep latency and subjective sleep quality. These findings may be relevant given that major depressive disorder has been reported in up to 56% of individuals with PNES, contributing to greater functional impairment and poorer outcomes [5,10].

Importantly, the clinical presentation of depression in PNES may differ from that observed in primary depressive disorders. In a comparative study, Martino et al. found that patients with PNES exhibited significantly higher levels of anxiety, alexithymia, and somatoform dissociation than individuals with mild-to-moderate major depressive disorder, along with a greater inability to verbalize emotions [44]. These features suggest that depressive symptoms in PNES are often embedded within broader processes of affective dysregulation and somatization. This interpretation is reinforced by Araújo Filho and colleagues, who reported not only high current and lifetime prevalence rates of depression in PNES patients (up to 80%) but also emphasized the role of unexpressed emotional distress and past trauma as contributing factors to seizure expression [45]. Moreover, adequate treatment of depressive symptoms has been associated with decreased PNES frequency, highlighting the central role of affective stabilization in the management of this population. A recent meta-analysis further confirms that depression is more prevalent and more often somatically expressed in PNES compared to epilepsy, and that interpersonal difficulties—rather than illness-related variables—appear to play a more central etiological role [46]. In this context, interventions that cultivate emotional awareness, reduce experiential avoidance, and foster embodied affect regulation—such as mindfulness-based protocols—may be particularly well-suited for this clinical population.

Sleep quality also showed a trend towards improvement, with a modest reduction in global PSQI scores and in components such as sleep latency and subjective sleep disturbance. These aspects are of particular interest in PNES, as patients in this population frequently report significantly impaired sleep quality, including difficulties initiating and maintaining sleep, non-restorative sleep, and excessive daytime fatigue [47]. These complaints are often more severe than those observed in patients with epilepsy or healthy controls. However, objective measures of sleep architecture—such as polysomnography—have yielded mixed results. Some studies have described alterations like reduced REM latency and increased REM percentage in PNES, resembling patterns seen in mood disorders, but others have failed to confirm consistent abnormalities [48]. This discrepancy may reflect phenomena such as paradoxical insomnia, somatization, or heightened interoceptive vigilance.

In contrast to typical findings reported in the literature, scores on the FFMQ “Describing” subscale decreased following the intervention. While mindfulness programs are generally associated with improvements in the ability to articulate inner experiences, patients with PNES frequently present with alexithymia, dissociative tendencies, and altered self-perception, which may complicate the relationship between awareness and verbal expression. Mindfulness practice may, in fact, initially heighten awareness of expressive limitations, leading to lower self-reported ability to verbalize emotions—a pattern previously described in alexithymic populations [49,50]. In this context, greater awareness of complex internal states could have temporarily amplified the perception of expressive difficulties, resulting in reduced scores. Similar paradoxical patterns have been observed in trauma-related and alexithymic samples, where increased introspection can exacerbate the recognition of expressive deficits [51,52]. Future mindfulness-based protocols might, therefore, be adapted to better support these patients by incorporating targeted strategies: preliminary evidence already suggests that mindfulness programs enriched with strategies for emotional labeling and expression can be beneficial. For example, mindfulness-informed exercises such as the “Emotion Mapping Activity” [53] or the integration of affect-focused practices like the “Affect Dyad” [54] have shown promise in enhancing emotional awareness and verbalization. Similarly, body-oriented mindfulness approaches have been found to reduce alexithymia by strengthening the connection between interoceptive signals and emotional understanding (e.g., recent body-based mindfulness trials) [50,55].

While overall interoceptive awareness did not significantly change, some MAIA subscales—especially Emotional Awareness and Self-Regulation—showed improvement trends. These areas are important for PNES patients, who mainly experience altered interoceptive sensibility rather than accuracy [56,57]. Individuals with functional seizures have been found to report lower levels of trust in bodily signals and greater difficulty disengaging from aversive sensations, as per the MAIA subscales Trusting and Not-Distracting [57]. This may reflect a form of interoceptive hypervigilance or distorted body-related insight, rather than a lack of bodily awareness per se, and could be consistent with the broader hypothesis that PNES symptoms arise from maladaptive attempts to regulate internal states that are poorly integrated at the cognitive-affective level [7,15]. While studies using behavioral measures such as heartbeat detection have often failed to demonstrate interoceptive deficits in PNES, patients consistently report heightened awareness of bodily discomfort paired with emotional confusion and alexithymic traits [56]. This difficulty in integrating internal experiences may also manifest as dissociative tendencies, which have long been associated with PNES and are thought to play a role in certain presentations of the disorder.

In the context, the presence of mild to moderate dissociation at baseline supports previous findings highlighting its clinical relevance in PNES. Dissociation in this population has often been interpreted as a defensive response to overwhelming affect or trauma, but recent evidence suggests a more nuanced view. While Myers et al. found that PNES patients scored higher on somatization compared to those with epilepsy, they did not differ significantly on dissociation measures, suggesting that dissociative traits may not be universally elevated across all PNES presentations [56]. However, their principal component analysis identified dissociation as part of a broader latent dimension involving somatization and demoralization, indicating a possible role for dissociation in specific PNES phenotypes. Other authors have emphasized the importance of considering the phenomenology of consciousness alteration in PNES. Roberts and Reuber argue that in some cases, seizures may reflect a dissociative shutdown linked to excessive emotional inhibition, while in others they may represent a direct, behavioral expression of dysregulated emotion, even in the absence of formal dissociation [57]. This variability may account for the differing results observed across studies, indicating a need for more detailed assessments of dissociative processes, as well as their association with emotional regulation styles. From a clinical perspective, these findings indicate that dissociation in PNES is unlikely to represent a single, unified process. Instead, it may be more appropriate to conceptualize dissociation as a spectrum of altered awareness and disruptions in self-regulation, with variable expression among patients.

Based on the findings discussed so far, patients with PNES exhibit a range of interconnected characteristics that are not confined to traditional diagnostic categories. These features suggest the relevance of a transdiagnostic treatment perspective, aimed at restoring access to bodily and emotional signals and promoting more adaptive regulatory capacities. Within this context, mindfulness-based interventions may have significant utility by promoting present-moment awareness, decreasing experiential avoidance, and strengthening embodied awareness. They may support a more integrated sense of self and improve emotional clarity, thereby reducing the need for maladaptive coping strategies such as dissociation or somatic symptom expression [15,17].

While this pilot study is constrained by its sample size and limited statistical power, it provides initial evidence indicating the potential clinical significance of mindfulness-based interventions for patients with PNES. Improvements were observed in mood, sleep, and emotional regulation, even in the absence of robust effects on seizure frequency. These changes align with the hypothesis that PNES symptoms are rooted in transdiagnostic mechanisms such as affective dysregulation, experiential avoidance, and altered body awareness. By addressing these core vulnerabilities, mindfulness-based approaches may represent a valuable adjunct to standard care. Moreover, preliminary observations of reduced seizure frequency, although not statistically significant, may reflect the downstream effects of enhanced emotional and bodily regulation fostered by mindfulness practice. The good acceptability and feasibility of the protocol, including its online adaptation, suggest that such interventions can be flexible and integrated into clinical settings. Future studies should confirm these findings with larger, controlled samples, ideally multicenter randomized controlled trials, and incorporate seizure diaries and objective biomarkers (e.g., EEG, HRV) together with longer follow-up assessments to evaluate symptom change. In addition, drawing from existing adaptations of mindfulness-based interventions, future protocols for PNES could integrate targeted strategies for emotional labeling, such as guided affect recognition, metaphor-based exercises, or structured journaling—alongside traditional mindfulness practices, to more effectively address expressive vulnerabilities that are common in this population.

### Study Limitations

This study has several methodological considerations that may affect the generalizability and interpretation of its results. The sample size is small, and a control group was not included, which substantially limits internal validity and generalizability of the results. In addition, without a control group, the observed changes could reflect non-specific factors such as natural symptom fluctuation, placebo or expectation effects, or concurrent treatments. Results—particularly those in the *p* = 0.05–0.10 range—should be viewed as exploratory. Additionally, follow-up assessments were not performed. All outcome measures, including “seizure” frequency, were based on patient self-report. This approach may limit reliability, particularly in PNES populations where altered self-perception, alexithymia, and dissociative features are common. Moreover, self-reported seizure frequency is subject to recall bias and may not accurately reflect clinical reality. The testing battery employed was intentionally limited to reduce participant burden, resulting in a narrower assessment of cognitive and emotional functioning. Although participants generally engaged well with the program, qualitative feedback revealed occasional difficulties with the meditative language and with sustaining practice between sessions. The university/hospital setting may also have influenced participants’ experience. Future studies should aim to enhance engagement and acceptability by providing more suitable and less institutional spaces, as well as simplified materials and structured support for home practice. They should also incorporate more objective and prospective measures—such as seizure diaries, ecological momentary assessment, or wearable devices—to improve accuracy and validity. These limitations represent critical weaknesses that prevent any definitive conclusions about efficacy; rather, the findings should be viewed as exploratory.

## 5. Conclusions

This pilot study indicates that a mindfulness-based intervention is feasible and acceptable for patients with PNES, including in an online format. Preliminary improvements were observed in depressive symptoms, sleep quality, and mindfulness skills, but these results must be interpreted with caution, given the small sample size and absence of a control group. Future research should confirm these findings through adequately powered multicenter randomized controlled trials, integrating objective biomarkers (e.g., EEG, HRV), seizure monitoring tools, and longer follow-up assessments. Refinements in delivery, such as more suitable settings and structured home-practice support, may further enhance acceptability. Overall, the present findings should not be interpreted as evidence of clinical efficacy but rather as preliminary indications supporting the need for further research on the potential utility of mindfulness-based interventions in PNES.

## Figures and Tables

**Table 1:** Summary of theoretical links between mechanisms implicated in PNES and potential targets of MBIs.

PNES Characteristics	Potential Target of Mindfulness Practice
Experiential avoidance	Non-judgmental acceptance and present-moment grounding
Altered sense of agency	Enhanced mind–body connection and interoceptive insight
Somatization	Increased emotional awareness and emotional regulation
Alexithymia	Improved recognition and labeling of emotions (observing)

**Table 2:** Sociodemographic characteristics of the sample. Mean age is reported with standard deviation and range.

Variable	Value
Gender	8 Males, 4 Females
Age (mean ± SD)	54 ± 6.6 years
Age range	42–63 years

**Table 3:** Pre–post comparison of psychological measures (N = 12). Means and standard deviations (M ± SD) are reported for baseline (Pre) and post-intervention (Post) assessments. Z and *p* values refer to Wilcoxon signed-rank tests. Effect sizes are reported as rank-biserial correlations (r) with 95% confidence intervals (CIs; lower and upper bounds), calculated in JASP using bootstrap resampling. Results with *p* < 0.05 were considered statistically significant; results with *p* = 0.05–0.10 were interpreted as preliminary, non-significant trends. MAIA = mean of subscales.

Domain (Measure)	Pre (M)	Post (M)	Dir.	Z	*p*	Effect Size (r)	95% CI for r	Significance
Depression (BDI-II)	14.50 ± 5.40	10.83 ± 4.93	↓	17	0.092	0.564	[−0.002, 0.856]	Trend
Sleep quality (PSQI)	10.50 ± 6.49	9.25 ± 5.41	↓	13.5	0.078	0.591	[0.011, 0.873]	Trend
Perceived Stress (PSS-10)	20.17 ± 8.96	18.42 ± 6.43	↓	23	0.373	0.282	[−0.337, 0.731]	No
Perceived Anxiety (STAI Y 1)	46.33 ± 10.17	42.50 ± NA	↓	28	0.424	0.303	[−0.341, 0.753]	No
Mindfulness (FFMQ total)	124.33 ± 19.15	117.67 ± 25.21	↓	18	0.11	0.397	[−0.217, 0.786]	No
Non Reactivity (FFMQ)	21.33 ± 5.80	19.75 ± 5.97	↓	23.5	0.266	−0.236	[−0.735, 0.429]	No
Non Judging (FFMQ)	27.67 ± 6.37	29.08 ± 6.91	↑	21	0.506	0.115	[−0.481, 0.639]	No
Observing (FFMQ)	20.42 ± 5.92	19.83 ± 5.87	↓	34.5	0.791	0.697	[0.191, 0.910]	No
**Describing** **(FFMQ)**	**26.92 ± 7.20**	**23.67 ± 7.74**	↓	**10**	**0.04**	0.564	[−0.002, 0.856]	**Yes**
Acting with Awareness (FFMQ)	27.75 ± 6.02	25.33 ± 6.11	↓	17	0.092	0.538	[−0.039, 0.846]	Trend
Interoception (MAIA total)	2.61 ± 1.94	3.14 ± 1.45	↑	9	0.109	−0.036	[−0.626, 0.580]	No
Noticing (MAIA)	2.54 ± 1.33	2.60 ± 1.17	↑	26.5	0.919	−0.127	[−0.679, 0.516]	No
Not Distracting (MAIA)	2.11 ± 1.04	2.44 ± 0.97	↑	24	0.719	−0.038	[−0.591, 0.539]	No
Not Worrying (MAIA)	2.91 ± 0.94	2.92 ± 0.75	↑	37.5	0.91	−0.273	[−0.739, 0.370]	No
Attention Regulation (MAIA)	2.10 ± 1.36	2.26 ± 1.18	↑	24	0.423	0.333	[−0.311, 0.768]	No
Emotional Awareness (MAIA)	3.80 ± 0.93	3.33 ± 1.00	↓	22	0.328	−0.530	[−0.851, 0.077]	No
Self-Regulation (MAIA)	2.12 ± 1.33	2.88 ± 1.13	↑	15.5	0.119	−0.167	[−0.669, 0.440]	No
Body Listening (MAIA)	1.87 ± 1.57	2.39 ± 1.33	↑	32.5	0.622	−0.600	[−0.891, 0.042]	No

## Data Availability

The data presented in this study are available on reasonable request from the corresponding author.

## Abbreviations

The following abbreviations are used in this manuscript:

PNES	Psychogenic Non-Epileptic Seizures
ICM	Integrative Cognitive Model
MBSR	Mindfulness-Based Stress Reduction
MBI	Mindfulness-Based Intervention
BDI-II	Beck Depression Inventory-II
PSQI	Pittsburgh Sleep Quality Index
PSS-10	Perceived Stress Scale—10 items
STAI-Y	State-Trait Anxiety Inventory—Form Y
FFMQ	Five-Facet Mindfulness Questionnaire
MAIA	Multidimensional Assessment of Interoceptive Awareness
DES-II	Dissociative Experiences Scale—II
METEO-Q	Meteoropathy Questionnaire
AOUP	Azienda Ospedaliero-Universitaria Pisana

## Appendix A

Example of Clinical Interview for PNES

**Seizure History**

- At what age did your seizures begin?
- Can you describe your very first seizure?
- How often do your seizures occur (per week/month)?
- How long does a typical seizure last?
- Do you experience any warning signs before the seizure?
- Are the episodes stereotyped (i.e., do they always present in the same way)?
- During episodes, what symptoms occur (e.g., motor activity, loss of consciousness, sensory changes, loss of urine, tongue biting/morsus)?
- Do you have the feeling that you can control your seizure in some way?
- What usually happens during the seizure (movements, sensations, loss of consciousness)?
- What do bystanders or family members observe during your seizures?
- What happens after the seizure (confusion, fatigue, rapid recovery)?
- Have you ever injured yourself during a seizure?

**Context and Triggers**

- In what situations do seizures mostly occur (alone, in public, at home)?
- Are your seizures more frequent during particular times (e.g., day/night, stressful periods)?
- Do you notice any relation with your sleep, medication use, or physical activity?

**Medical and Neurological History**

- Do you have a family history of epilepsy?
- Did you have previous neuroimaging (CT, MRI) performed to exclude organic causes?
- Have you been diagnosed with epilepsy or any other neurological condition in the past?
- Have you undergone EEG or video-EEG monitoring? If yes, what were the results?
- What treatments have you received for your seizures (medications, psychotherapy, hospitalization)?
- Any significant head injuries, neurological illnesses, or known medical conditions?

**Psychological and Psychiatric History**

- Have you ever experienced psychological trauma (abuse, accidents, losses)?
- Have you ever been diagnosed with a mental health condition (anxiety, depression, PTSD)?
- How do you usually cope with stress?
- Do you have a history of self-harm or suicidal thoughts?

**Personal and Social History**

- Tell me about your current living situation and social support.
- How has your education and work life been affected by the seizures?
- How do your seizures impact your daily functioning and quality of life?
- How do people around you (family, colleagues) react to your seizures?
